# Characterization and Immunomodulation of Canine Amniotic Membrane Stem Cells

**DOI:** 10.2147/SCCAA.S237686

**Published:** 2020-05-07

**Authors:** Alessandra de Oliveira Pinheiro, Valéria M Lara, Aline F Souza, Juliana B Casals, Fabiana F Bressan, Paulo Fantinato Neto, Vanessa C Oliveira, Daniele S Martins, Carlos E Ambrosio

**Affiliations:** 1 Department of Veterinary Medicine, Faculty of Animal Science and Food Engineering, University of São Paulo, Pirassununga, São Paulo, Brazil; 2 Private Veterinary Practice, Pirassununga, São Paulo, Brazil

**Keywords:** canine stem cells, immunomodulation, fetal annexes

## Abstract

**Purpose:**

Amniotic membrane stem cells have a high capacity of proliferation, cell expansion, and plasticity, as well as immunomodulatory properties that contribute to maternal-fetal tolerance. Owing to the lack of research on human amniotic membrane at different gestational stages, the canine model is considered ideal because of its genetic and physiological similarities. We aimed to characterize the canine amniotic membrane (CAM) cell lineage in different gestational stages and evaluate the expression of immunomodulatory genes.

**Materials and Methods:**

Twenty CAMs from early (20–30 days) (n=7), mid- (31–45 days) (n=7), and late gestation (46–63 days) (n=6) stages were studied. The cell features were assessed by cell viability tests, growth curve, colony-forming units, in vitro differentiation, cell labeling for different immunophenotypes, and pluripotent potential markers. The cells were subjected to RT-PCR and qPCR analysis to determine the expression of *IDO, HGF, EGF, PGE2*, and *IL-10* genes.

**Results:**

CAM cells exhibited a fibroblastoid morphology and adherence to plastic with an average cell viability of 78.5%. The growth curve indicated a growth peak in the second passage and we obtained an average of 138.2 colonies. Osteogenic, chondrogenic, and adipogenic lineages were confirmed by in vitro differentiation assays. Cellular immunophenotyping experiments confirmed the presence of positive mesenchymal markers (CD90 and CD105) and the low or negative expression of hematopoietic markers (CD45 and CD34). Qualitative analysis of the immunomodulatory functions indicated the expression of the *IDO, HGF, EGF5*, and *PGE2* genes. When stimulated by interferon-gamma, CAM cells exhibited higher IDO levels throughout gestation.

**Conclusion:**

The CAMs from different gestational stages presented features consistent with mesenchymal stem cell lineage; better results were observed during the late gestation stage. Therefore, the gestational stage is a key factor that may influence the functionality of therapies when using fetal membrane tissues from different periods of pregnancy.

## Introduction

Mesenchymal stem cells (MSCs) are characterized by a multipotent cell lineage with a high capacity of differentiation into different cell types, which can execute trophic, paracrine, and immunomodulatory functions in other cells based on the microenvironment.^1^,^2^

In regenerative medicine, MSCs derived from fetal annexes such as amniotic fluid, amniotic membrane, and umbilical cord blood and vessels have previously been isolated and well-characterized in humans^3^ and other species, such as dogs,^4^–^7^ cats,^8^,^9^ bovines,^10^ sheep,^11^,^12^ horses,^13^ rats,^14^ rabbits,^15^ and ducks,^16^ suggesting the advantages of using it as raw material for the creation of cell banks.^17^–^20^

Immunomodulatory activities occur through direct contact between the MSCs and tissues or through paracrine interaction mediated by interferon-gamma (IFN-γ), produced by the body’s immune cells, which act on natural killer cells, monocytes, neutrophils, and macrophages. Some of the T-helper lymphocytes, cytotoxic T lymphocytes, and B lymphocytes secret soluble factors such as TGF-β, interleukin-10 (IL-10), interleukin-6 (IL-6), indoleamine-2,3-dioxygenase (IDO), prostaglandin E2 (PGE2), and soluble human leukocyte antigen-G5 (sHLA-G5), or interact by cell-cell reactions,^21^ in addition to preventing the expression of proinflammatory cytokines such as IFN-γ and tumor necrosis factor-α (TNF-α).^22^,^23^

Based on the significant immunomodulatory properties of MSCs and the limited number of studies on canine amniotic membrane (CAM) stem cells from different gestational stages, we aimed to characterize the CAM stem cells derived from different gestational stages and to determine the in vitro immunomodulatory potential and to establish a cell line that can be employed in the treatment of several diseases that affect domestic animals.

## Materials and Methods

This study was approved by the Ethical Committee on Animal Use (Comissão de Ética no Uso de Animais) of the Faculty of Animal Science and Food Engineering of the University of São Paulo (protocol #4,598,140,116). We follow the international guide for use of dogs in experiments. https://www.ccac.ca/Documents/Standards/Guidelines/Vol2/dogs.pdf

### Isolation and Culture of Stem Cells from CAM

**Figure 1:**
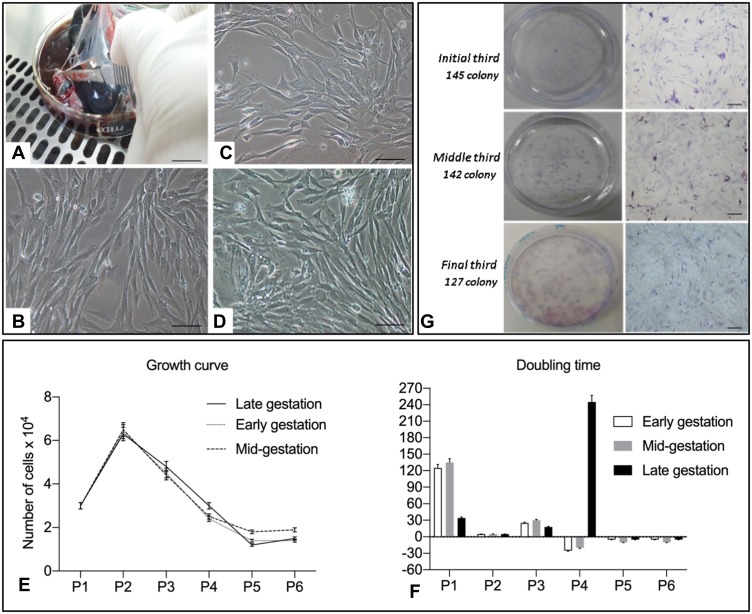
(**A**) Cell collection from the amnion. (**B**) Cell culture from passage 1 of amniotic membranes from the initial third 20x canine fetuses (Bar: 50 μm). Cell culture from passage 1 of amniotic membranes from the middle third of canine fetuses (Bar: 50 μm); (**C**, **D**) cell culture from passage 1 of amniotic membranes from the final third of canine fetuses (Bar: 50 μm); (**E**) graphical representation of the growth curve profile of canine amniotic membrane (CAM) stem cells; (**F**) doubling time graph indicating the number of days required for doubling the number of CAM cells; (**G**) colony-forming unit (CFU) test of CAM cells from the three gestational stages stained by Giemsa (Bar: 100 µm).

Twenty CAMs from different gestational stages, early (20–30 days) (n=7), mid- (31–45 days) (n=7), and late gestation (46–63 days, due parturition) (n=6), were collected from the uteri of pregnant mixed-breed domestic dogs. The amniotic membrane fragments (Figure 1A) were washed and digested with 1 mL of Collagenase type I (Sigma, St. Louis, USA). The cells were cultured in plastic plates using αMEM medium (Gibco, New York, USA) supplemented with 15% fetal bovine serum (FBS, Invitrogen, Carlsbad, USA) and 1% penicillin/streptomycin (Gibco, New York, USA), and were incubated at 38.5°C, 5% CO_2_, and maximum humidity.

### Analysis of Cell Viability, Growth Curve, Doubling Time, and Colony-Forming Assays

To assess the cell viability, the cells were separated in triplicate at a density of 4x10^4^ cells/cm^2^ and frozen. After thawing, the cells were stained with trypan blue (1:1, Sigma-Aldrich, Brazil) to assess cell viability. The viable cells were counted using a hemocytometer and a Newbauer chamber. The cells were frozen for 24 h at −80°C in Mr. Frosty Freezing container (Sigma-Aldrich, Brazil) and stored in liquid nitrogen. After freezing and thawing, the cells were counted using a Newbauer camera.

The growth curve was estimated from the triplicate and the cells were plated and counted using a hemocytometer; 3x10^4^ cells were plated on 35-mm plates and were maintained in an incubator at 38.5°C. The cells were counted every 96 h and were subcultured at the same density until passage 5. The formula Ct/Cd was used to calculate the doubling time, where Ct represents the culture time between passage n and passage n+1 and Cd represents cell doubling. Cell doubling was calculated using the formula Cd=ln (nf/ni) ln2, where nf and ni represent the number of harvested and seeded cells, respectively. Doubling time was calculated, as described by Vidane et al.^9^

For the colony-forming unit assays, 1x10^4^ cells were plated in 90-mm Petri dishes using the culture medium. The colony formation assays were conducted over 13 days, and the culture medium was changed every 3 days. After 13 days, colonies were observed and adherent cells were fixed in 4% paraformaldehyde (Sigma, St. Louis, USA) and stained for 15 min using Giemsa 0.1% stain.

### In vitro Differentiation Assays (Osteogenic, Adipogenic, and Chondrogenic)

All differentiation assays were performed during passage 2. To promote osteogenic and adipogenic differentiation, 1x10^5^ were cultured in StemXVivo™ Human/Mouse Osteogenic/Adipogenic Base Media (R&D, Minneapolis, USA) supplemented with penicillin-streptomycin; after 21 days, the plates were fixed with 4% paraformaldehyde and stained by Alizarin Red and Sudan Black, respectively. For chondrogenic differentiation, 3x10^5^ cells were placed in Falcon tubes with 5 mL of StemXVivoTM Human/Mouse Chondrogenic (R&D). After 3 days, the cells were resuspended in 2 mL of differentiation medium (R&D). After 21 days, the pellets were fixed, stained using Alcian Blue and Masson Trichrome, and analyzed.^9^

### Flow Cytometry

Flow cytometry was used to analyze the reactivity of mesenchymal (CD90 and CD105) and hematopoietic (CD34 and CD45) specific markers (Table 1). Cells were taken from passage 2 (1x10^5^ cells) and incubated for 20 min at 4°C with each antibody. Samples were analyzed using the flow cytometer FACSAria-BD Cell Sorter supported by DiVa software V.6.1.2 (BD Biosciences, San Jose, CA, USA).

**Table 1:** Specifications of the Primary and Secondary Antibodies Used for the Characterization of Mesenchymal and Hematopoietic Markers

Primary Antibodies	Isotype	Company	Catalogue Number	Species	Monoclonal Polyclonal	Species Reactivity
CD105 Dilution pure	IgG2b	Abcam	156,756	Mouse	Mono	Mouse, rat, dog, human, monkey
CD34 Dilution 1:100	IgG1	eBioscience	12-0340-42	Mouse	Mono	Dog
CD90 Dilution 1:100	IgG2b	eBioscience	12-5900-42	Rat	Mono	Dog
CD45 Dilution 1:100	IgG2b	eBioscience	11-5450-42	Rat	Mono	Dog
Secondary antibody						
Goat anti-mouse Dilution 1:50	IgG	Dako	F0479	Mouse	Poly	Mouse

### Evaluation of the Immunomodulatory Response

CAM cells from passage 2 at different gestational stages were cultured in triplicate at a density of 5x10^5^. The cells were treated with 200 ng/mL IFN-γ (R&D) for 72 h, as described by Saulnier et al 24 and Russel et al.^25^ After this period, the cells were evaluated for *IDO, HGF, EGF, PGE-2*, and *IL-10* expression.

### Real-Time Polymerase Chain Reaction (qPCR) for Evaluation of Immunomodulatory and Pluripotency Genes

Total RNA was extracted from the CAM cells from passage 2 using the TRIzol LS reagent (Life Technologies, Carlsbad, USA) following the manufacturer’s protocol. The total cellular RNA concentration was quantified by the Nanodrop 1000 spectrophotometer (Nanodrop Technologies, Wilmington Delaware, USA). For cDNA synthesis, the mRNA was reverse transcribed using the Enzyme Reverse Transcriptase superscript III kit (Invitrogen) according to the manufacturer’s specifications. Gene expression was assessed by qPCR (Step One Plus Real-Time PCR Systems, Life Technologies). The reactions were performed using a commercial assay system (PowerUpTM SYBR^®^ Green PCR Master Mix, Applied Biosystems^®^, Carlsbad, USA) with *OCT4* and *SOX2* (pluripotency genes) and *IDO, HGF, EGF, PGE-2*, and *IL-10* (immunomodulatory genes) as target genes of interest. The *18S* gene, a housekeeping gene, served as the control. The primer sequences are presented in Table 2. The reaction conditions consisted of 40 cycles at an annealing temperature of 60°C were quantified by normalizing the signals to the 18S signals using the 2^−ΔCT^ method.

**Table 2:** Sequence of Primers Used in the Evaluation of the Expression of Pluripotency and Immunomodulatory Genes

Gene	Sequence (5ʹ-3ʹ)	Primer	Accession no.	bp	References
*IDO*	GCTGGGTCTGCCTCCTATTC GCAGTCTCCACCAGGAAACC	Forward Reverse	NC_006598.3	126	[33]
*EGF*	CTATGGCCCTCAAGGATGGTG GCAGCCTTGCTCTGTGTCCTTA	Forward Reverse	NC_006614.3	124	[29]
*HGF*	GGCTACTGCTCCCAAATTCCA CCCACATTGAACATGTTAGTCCAGA	Forward Reverse	NC_006600.3	123	[29]
*IL-10*	AGCACCCTACTTGAGGACGA ACTGGATCATCTCCGACAGG	Forward Reverse	NC_006589.3	198	[36]
*IL-6*	GAACTCCCTCTCCACAAGC TTCTTGTCAAGCAGGTCTCC	Forward Reverse	NC_006596.3	325	[42]
*PGE-2*	CTGTCATCACCGGCCAAGT CCTGGTCACTCCGGCAATA	Forward Reverse	NC_006590.3	99	[49]
*OCT-4*	GCAGTGACTATTCGCAACGA ATTTGAATGCATGGGAGAGC	Forward Reverse	NC_006594.3	-	[50]
*SOX-2*	CCCACCTACAGCATGTCCTA GGAGTGGGAGGAGGAGGTAA	Forward Reverse	NC_006616.3	-	[7]
*18S*	CCTGCGGCTTAATTTGACTC CTGTCAATCCTGTCCGTGTC	Forward Reverse	NC_006605.3	65	[48]

### Reverse Transcriptase-Polymerase Chain Reaction (RT-PCR) for Evaluation of Immunomodulatory Genes

The CAM cells from passage 2 were subjected to RT-PCR analysis using the GoTaq^®^ Green Master Mix Kit (Promega^®^, Madison, USA); the Applied Biosystems Veriti 96-well Thermal Cycler (Applied Biosystems^®^) was used for processing the reactions. Sequences were amplified under the following conditions: 95°C for 2 min, followed by 30 cycles of 30 s at 95°C, 30 s at 56.5°C, and 1 min at 72°C, followed by a final extension at 72°C for 5 min. At the end of the reaction, the products were analyzed in 2% agarose gel (Sigma, Carlsbad, USA) stained in solution containing SYBR^®^ safe DNA Gel Stain (Invitrogen) to detect the *IDO, HGF, EGF, PGE-2*, and *IL-10* genes and were compared with 100 bp (1 kb Plus DNA Ladder, Invitrogen) markers. The endogenous *18S* gene (Table 2) was used as a reference.

### Statistical Analysis

The data obtained from the experimental procedures were analyzed using the program Graphpad Prism^®^, with prior verification of residue normality by the Shapiro–Wilk test. The variables that did not meet the statistical assumptions were subjected to the logarithmic transformation *[Log (X + 1)]*. The original or transformed data were subjected to Analysis of Variance (p<0.05) as required. The time and treatment effects were evaluated by the Tukey-Krammer’s test. Effects were considered significant for p<0.05.

## Results

### Isolation and Culture of Stem Cells from CAMs

CAM cells from different gestational stages were successfully cultured, as shown in Figure 1B–D. Cells in primary culture adhered to the plastic surface after a 48-h period and a heterogeneous population of polygonal and fibroblastoid cells was formed.

### Cryopreservation and Cellular Susceptibility

After cryopreservation, the CAM cells were thawed and an average viability of 83.3% was observed in cells from the early gestation stage. In the mid- and late gestation stages, the average viabilities were 80.1% and 75.22%, respectively.

### Growth Curve

After a 96-h period, a confluence of 80% was observed in the culture plates. After repeated passages, the growth curve indicated high development of cells at the beginning of procedures, and notably, a predominance of proliferation was noted in passage 2, while a gradual reduction of the proliferation was observed in passage 5 (Figure 1E). The Doubling time increased with each additional passage. The gradual reduction in cell proliferation rate was in accordance with successive increments in passages (Figure 1F).

### Colony-Forming Assays

Colony formation was observed after 9, 10, and 13 days in CAM cells from early, mid-, and late gestation, with 145, 142.5, and 127.3 colonies observed, respectively (Figure 1G).

### In vitro Differentiation Assays

**Figure 2:**
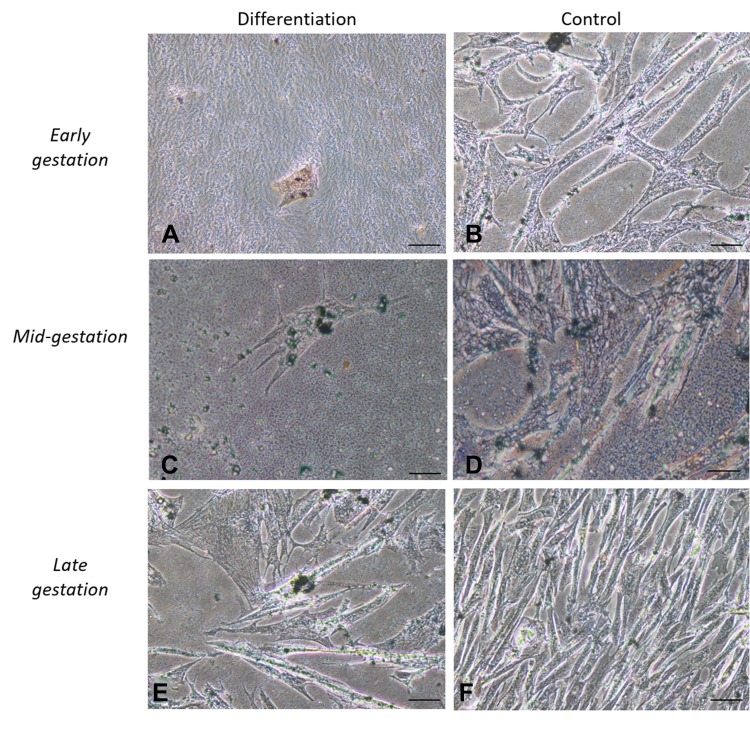
In vitro differentiation adipogenic. Legend: In vitro differentiation in the adipogenic line of CAM stem cells. (**A**), (**C** and **E**) Intracellular Sudam black stained, it was possible to identify lipid droplets and followed by induction of adipogenic differentiation (Bar: 50µm); (**B)**, (**D** and **F**) negative controls (Bar: 50µm).

**Figure 3:**
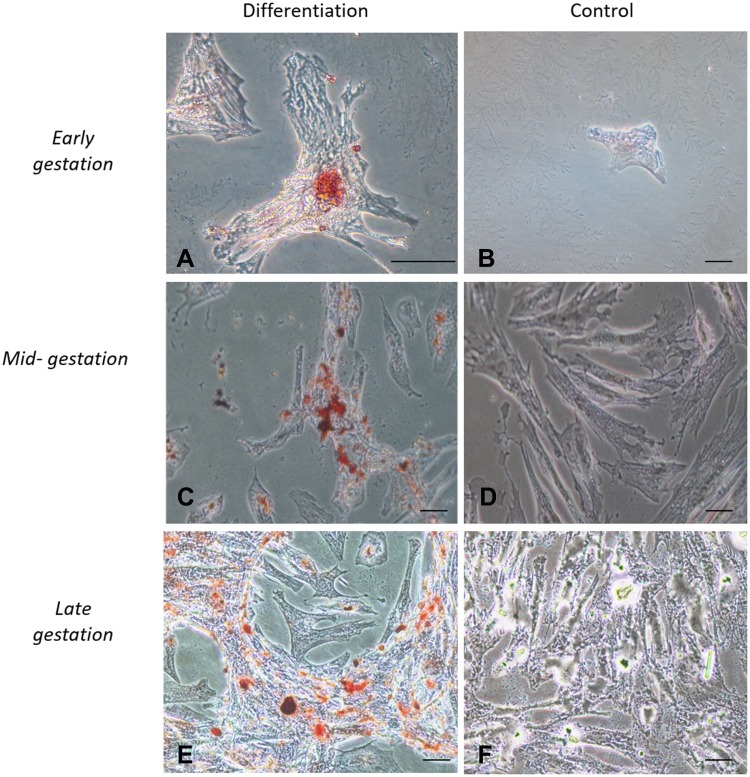
In vitro differentiation osteogenic. Legend: In vitro differentiation in the osteogenic lines of CAM stem cells. (**A**), (**C** and **E**) Deposition of extracellular calcium followed by osteogenic differentiation stained by Red Alizarin (Bar: 50µm); (**B**), (**D** and **F**) Negative controls (Bar: 50µm).

**Figure 4:**
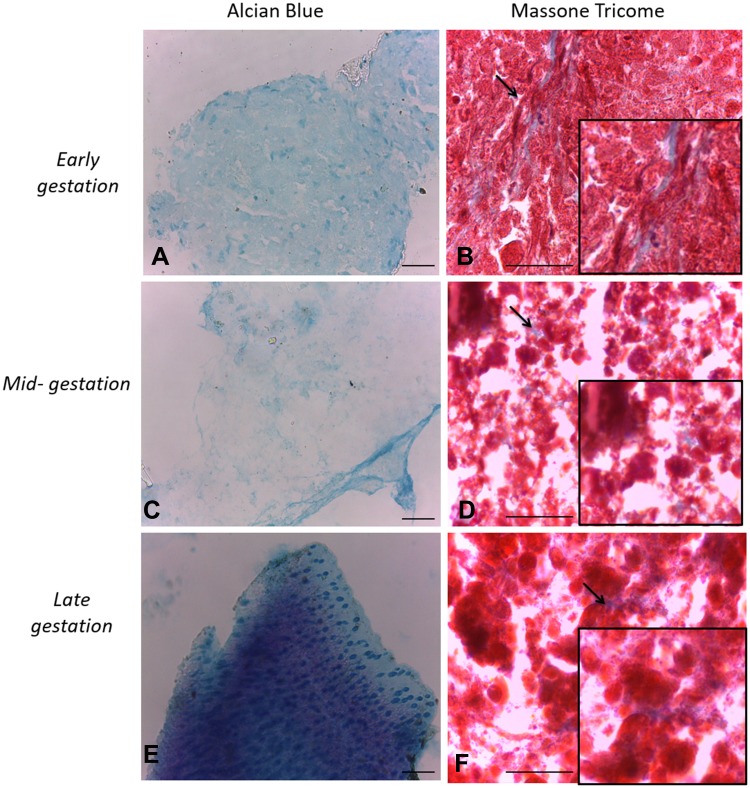
In vitro differentiation chondrogenic. Legend: In vitro differentiation in the chondrogenic lines of CAM stem cells. (**A**), (**C** and **E**) Collagen fibers of pellet culture stained in blue. Induction of chondrogenic differentiation stained by Alcian blue (Bar: 50µm); (**B**), (**D** and **F**) note the induction of chondrogenic differentiation stained Masson’s trichrome reaction (Bar: 50µm).

Cells cultured in the adipogenic differentiation medium exhibited morphological changes, with formation of intracytoplasmic vacuoles. Cells cultured in the osteogenic differentiation medium exhibited the deposition of extracellular amorphous mineral material. After being cultured in the chondrogenic differentiation medium, collagen fibers stained in blue were observed (Figures 2–4).

### Flow Cytometry

**Figure 5:**
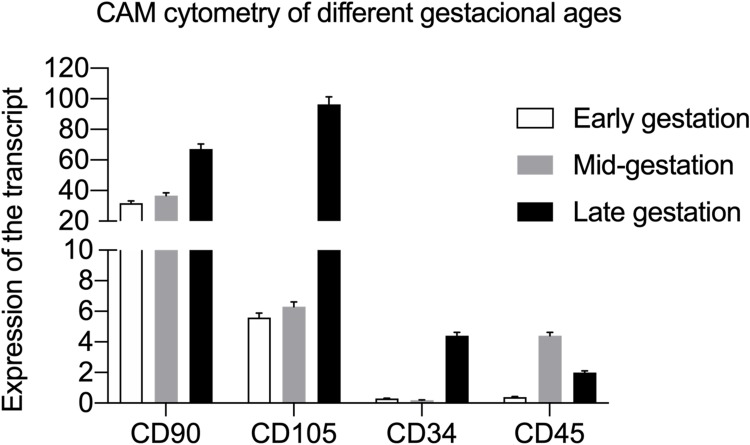
Quantification of the expression of mesenchymal (CD90 and CD105) and hematopoietic markers (CD45 and CD34) in CAM cells.

Early gestation CAM cells exhibited expression of CD90 (31.7%) and CD105 (5.6%) and low labeling of CD34 (0.3%) and CD45 (0.4%). CAM cells from the mid-gestation stage exhibited positivity toward the CD90 (36.7%) and CD105 (6.3%) markers, with low labeling of CD34 (0.2%) and CD45 (0.4%). CAM cells from the late gestation stage exhibited positivity toward CD90 (67.1)% and CD105 (96.4%) markers, with low labeling of CD34 (4.4%) and CD45 (2.0%) (Figure 5).

### Real-Time Polymerase Chain Reaction (qPCR) for Evaluation of Immunomodulatory and Pluripotency Genes

There was no expression of the *OCT-4* transcript in the CAM cells from the three gestational stages. There was a low expression of the *SOX-2* transcript at different stages, with a variability of abundance between gestational stages (p<0.05%).

**Figure 6:**
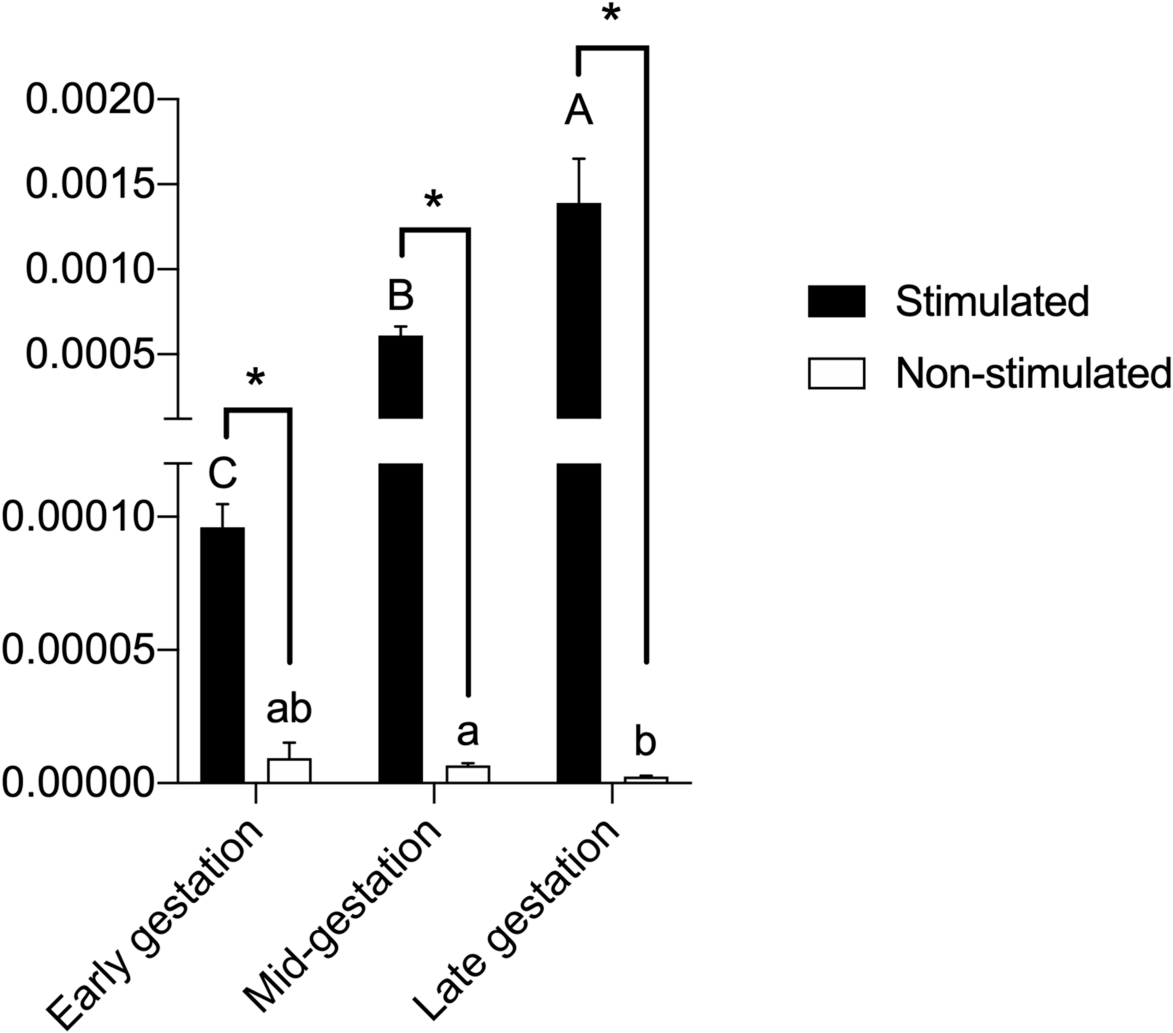
Legend: *IDO* expression in CAM cells stimulated with gamma-interferon (IFN-γ), or in non-stimulated cells. ^A–C^Uppercase letters indicate significant differences between different gestational stages in stimulated CAM cells. ^a,b^Lowercase letters indicate significant differences between different gestational stages in unstimulated CAM cells. * indicates significant differences between stimulated or unstimulated cells from the same gestational stage.

In the *IDO* transcript analysis, there was a significant difference (p<0.05) between the stimulated and unstimulated cells from each gestational stage, with higher expression in the stimulated cells. A significant difference (p<0.05%) was observed between the stimulated cells from early gestation and other gestational stages. Unstimulated CAM cells from late gestation were significantly different from CAM cells from mid-gestation, with lower expression in cells from late gestation (Figure 6). The expression of *HGF, EGF, IL-10*, and *PGE2* transcripts was not observed in any of the three gestational stages.

### Reverse Transcriptase-Polymerase Chain Reaction (RT-PCR) for Evaluation of Immunomodulatory Genes

**Figure 7:**
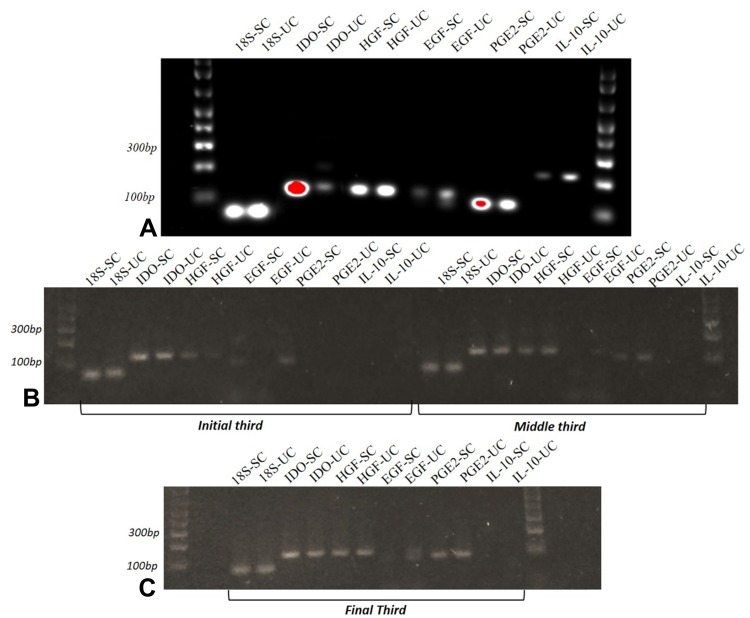
Electrophoresis of cytokines and growth factors. Legend: (**A**) Stimulated and non-stimulated canine bone marrow cells; (**B**) stimulated and non-stimulated CAM cells from early and mid-gestation; (**C**) stimulated and non-stimulated CAM cells from mid- and late gestation.

Analysis of the RT-PCR results revealed the expression patterns of *IDO, HGF, EGF, PGE2*, and *IL-10* transcripts in CAM cells from the three different gestational stages and from canine bone marrow, as shown in Figure 7.

## Discussion

MSCs are present in a majority of adult tissues and have a high proliferative capacity. In comparison to corresponding information from human and mouse lineage, there is limited information on the biology and function of canine MSCs in veterinary medicine. This lack of knowledge prevents the development of evidence-based studies using canine MSCs.^25^

In this study, we successfully established the CAM MSC line from early, mid-, and late gestation, based on the methodology described by Lange-Consiglio et al^13^ in the equine model. Initially, the CAM cells were isolated and subjected to primary culture, and they appeared to be proliferative and heterogeneous, with the presence of polygonal and fibroblastoid cells.^8^,^13^,^24^ After the primary culture, the cells displayed a fibroblastoid morphology with adhesion to the plastic surface, which is one of the characteristics of MSCs.^1^,^6^,^11^,^26^

Our observations from the growth curve analysis indicated a cell growth peak in the second passage, followed by a decline in proliferation in successive passages, corroborating with previous results.^9^,^27^ This decline was observed in MSCs from amniotic fluid, umbilical cord blood, and umbilical cord matrix as well, indicating a proliferation potential limited to the first passage.^4^,^28^ In contrast, Park et al reported an increasing cellular proliferation rate in amniotic membrane cells between the third and the twentieth passages, without a declining growth curve. The doubling time of CAM cells increased in terms of number of days required for cell duplication, a phenomenon observed in MSCs that undergo aging and quiescence as the number of passages progress.^5^,^9^,^24^,^27^

The three gestational stages exhibited differentiation capacity in adipogenic, chondrogenic, and osteogenic lines. The differentiation capacity is cited as one of the attributes necessary for stem cells to be classified as MSCs.^1^,^4^,^5^,^9^,^11^,^13^,^24^,^29^-^32^

The cellular immunophenotyping results revealed the expression of mesenchymal markers (CD90 and CD105) and non-expression of hematopoietic markers (CD45 and CD34) in CAM cells from different gestational stages. This was anticipated since the tested cells were not associated with the hematopoietic lineage. These results are consistent with findings from studies involving CAM cells that characterized MSC markers (CD29, CD44, CD90 CD105 and CD166) and negative markers for immune cells (CD3, CD11c, CD28, CD38 and CD62L), hematopoietic cells (CD34 and CD45), and platelets (CD41).^5^,^7^,^13^,^24^ Additionally, Saulnier et al^24^ and Cardoso et al^7^ reported a low expression of the CD105 marker in CAM cells.

Cells from the fetal annexes present certain advantages owing to their potential, since these can preserve embryonic characteristics by maintaining the pluripotency of original tissues.^18^,^24^,^33^ In our study, we reported low expression of *SOX2* and no expression of *OCT4* in the three gestational stages. Our results corroborated with those reported by Saulnier et al,^24^ wherein the noted low expression of *SOX2* gene and no expression of *OCT4* in CAMs, and in canine placenta and umbilical cord matrices. In contrast to our results, *OCT4* gene expression was reported in CAM cells in another study.^4^–^7^ Mauro et al^11^ reported the expression of *OCT4* in passage 1 sheep cells, with significant reduction after the passage 6. Conversely, Filioli Uranio et al^4^ reported a reduction in *OCT4* expression between passage 1 and 2. Due to the diversity of results described in the literature, more tests should be conducted on the pluripotency of CAM.

To date, this is the first study to investigate the immunomodulatory response of CAM stem cells at different gestational stages through the expression of soluble factors such as *IL-10, IDO, HGF, EGF*, and *PGE2*. A limitation in this field is the absence of studies evaluating cells from canine fetal annexes throughout gestation.

The *IDO* gene, identified in some species, plays a central role in the study of in vitro immunomodulation, acting in tryptophan catabolism and inhibition of T-lymphocyte proliferation.^34^ Stimulation with mitogens such as INF-γ, TNF-α, interleukin 1A (IL-1A), or interleukin 1β (IL-1β) enhances the expression of immunomodulatory genes.^35^–^37^ In our results, we observed a higher expression of *IDO* in cells stimulated with INF-γ, irrespective of the gestational stage. Saulnier et al 24 reported that CAM, placenta, and umbilical cord matrix stimulated with IFN-γ exhibited higher *IDO* expression than unstimulated cells from the same tissues.

Munn et al^38^ and Mellor et al^39^ highlight the significance of IDO during gestation, since pregnant mice exposed to IDO-inhibiting drugs were unable to maintain gestation. Likewise, in unstimulated CAM cells, IDO expression was lower in late gestation when compared to that in mid-gestation. Maternal-fetal recognition and placentation take place during the early to mid-gestation stages. Therefore, a higher expression of IDO, and consequently the inhibition of the local immune system against the allogenic fetus seems to be essential to initiation and maintenance of placentation, as it is to gestation. On the contrary, in late gestation, lower expression of IDO is noted as the immune system appears to be linked to parturition, and hence, to the release of the fetal membranes as well.

The results from IFN-γ stimulation experiments confirm the findings reported by Saulnier et al,^24^ since CAM cells exhibit increased IDO expression levels in the presence of inflammatory stimulation. Therefore, the late gestation CAM cells should display significant immunomodulatory potential prior to inflammatory stimulation.

Quantitative evaluation of *HGF, EGF, PGE2*, and *IL-10* expression in CAM cells yielded negative results. Conversely, immunomodulation-related soluble factors, such as IGF-1, bFGF, IL-10, TNFα, TGF-β, PGE2,^40^ HGF and EGF,^40^,^41^ have been identified in human amniotic membrane epithelial cells and MSCs. Kang et al^42^ were the first to describe the immunomodulatory potential of canine MSCs, demonstrating the expression of *TGF-β, IL-6, IL-8, CCL2, CCL5, EGF, HGF* and the non-expression of *IL-10* genes in adipose tissue cells. In canine adipose and bone marrow cells, lymphocyte activation was suppressed by the cyclooxygenase and *TGF-β* pathways, as well as by the commonly known pathways mediated by *NO* or *IDO*.^43^ In addition, Lee et al^28^ reported that BM-MSCs were able to inhibit leukocyte proliferation and highlighted *PGE2* as a potential antiproliferative factor.

This study involved the assessment of the immunomodulation of CAM by exclusive IFN-γ stimulation. Rossi et al^44^ reported that the immunological stimulation of human amniotic membrane cells is induced by soluble molecules and cell-to-cell contact. Di Nicola et al^45^ reported that the immunomodulatory effect on bone marrow cells is mediated exclusively by soluble factors. In contrast, Krampera et al^35^ reported that the immunomodulatory effect on rodent MSCs appeared to be predominantly mediated by cell-to-cell contact. Therefore, the diversity of results on the immunomodulation of MSCs may be directly related to the tissue being studied or the mechanisms inherent to each species,^46^ such as in dogs.^47^

## Conclusions

This is the first study that compared the cellular characterization and gene expression patterns linked to immunomodulation in CAMs from different gestational stages. We revealed that CAM cells undergo an immunophenotypic and immunomodulatory transformation during the gestational period, with more satisfactory results observed in cells from the late gestation stage. These results suggest a distinct clinical impact on new avenues of research. Owing to the scarcity of data confirming the pathways involved in the immunomodulatory response in dogs, we conclude that a better understanding of CAM-mediated immunosuppression is necessary for future clinical applications, and this may serve as a strategy in the treatment of several immune-mediated diseases.

## Abbreviations

CAM, canine amniotic membrane; IFN-γ, gamma-interferon; MSCs, mesenchymal stem cells.

## Data Sharing Statement

All data generated and/or analyzed during this study are included in this published article.
